# Efficacy of Levothyroxine Sodium Soft Gelatin Capsules in Thyroidectomized Patients Taking Proton Pump Inhibitors: An Open-Label Study

**DOI:** 10.1089/thy.2023.0382

**Published:** 2023-12-07

**Authors:** Issac Sachmechi, Kathryn Jean Lucas, Larry D. Stonesifer, John Fitzgerald Ansley, Paul Sack, Francesco S. Celi, Claudia Scarsi, Gabriele Lanzi, Leonard Wartofsky, Kenneth D. Burman

**Affiliations:** ^1^ Mount Sinai Services—NYC Health+Hospitals/Queens, Jamaica, New York, USA.; ^2^ LucasResearch, Inc., Diabetes & Endocrinology Consultants, Morehead City, North Carolina, USA.; ^3^ Larry D Stonesifer, MD, Inc., Federal Way, Washington, USA.; ^4^ Carolina Ear Nose and Throat Clinic CENTRI, Inc., Orangeburg, South Carolina, USA.; ^5^ MedStar Union Memorial Hospital, Baltimore, Maryland, USA.; ^6^ Division of Endocrinology and Metabolism, Department of Medicine, UConn Health, Farmington, Connecticut, USA.; ^7^ IBSA Institut Biochimique SA, Lugano, Switzerland.; ^8^ MedStar Health Research Institute, Washington, District of Columbia, USA.; ^9^ Endocrine Section, MedStar Washington Hospital Center, Washington, District of Columbia, USA.

**Keywords:** hypothyroidism, levothyroxine, capsules, proton pump inhibitors, Tirosint, antacids

## Abstract

**Background:**

Treatment with proton pump inhibitors (PPIs) and antacids affects the gastrointestinal absorption of levothyroxine sodium (LT4) tablets. Patients with hypothyroidism taking LT4 and PPIs or antacids, thus, require appropriate monitoring. The objective of this study was to determine whether a soft gelatin capsule of LT4 (Tirosint^®^) would obviate the effect of PPIs on LT4 absorption. The objective was achieved by assessing the effects of a switch from a conventional LT4 tablet form to the same dose as soft capsules in thyroidectomized patients on treatment with LT4 and PPIs.

**Methods:**

Patients with history of hypothyroidism due to total thyroidectomy on stable treatment with LT4 tablets, and with gastrointestinal disease treated with PPIs, were switched to a 12-week treatment with Tirosint at the same dose of the LT4 tablets, while maintaining treatment with PPIs. Serum thyrotropin (TSH) levels were the primary endpoint of the study. Secondary efficacy endpoints were: serum levels of free thyroxine (fT4), total thyroxine (TT4), free triiodothyronine (fT3), total triiodothyronine (TT3), creatine-phosphokinase (CPK), sex-hormone binding globulin, ferritin, angiotensin converting enzyme, and a lipid panel.

**Results:**

Forty-seven patients (36 females and 11 males, mean age 55.4 years) were enrolled and 45 of them completed the study (2 patients withdrew consent). During treatment with Tirosint, mean TSH levels demonstrated a statistically significant decrease (mean changes from baseline: −0.32 mIU/L at week 6 and −0.68 mIU/L at week 12) and concomitant increases in thyroid hormone (TH) levels from baseline to week 12, which were statistically significant for fT3 and TT3 (mean changes from baseline: 0.26 pmol/L and 0.10 nmol/L, respectively). Significant decreases of serum low-density lipoprotein, total cholesterol, and CPK levels were observed at week 12. No signs/symptoms arose during the study that could be specifically correlated to either hypo- or hyperthyroidism.

**Conclusions:**

In thyroidectomized patients taking PPIs and replacement LT4, a switch from conventional LT4 tablets to LT4 soft capsules at the same dose was associated with a significant decrease in TSH and increase in TH, indicating that LT4 absorption may be less affected by PPIs when given in the form of soft capsules. Clinical Trial Registration: NCT03094416.

## Introduction

Levothyroxine sodium (LT4) is the treatment of choice for hypothyroidism. In patients with no significant comorbidities, the recommended LT4 full replacement dose is about 1.6–1.7 μg/kg/day.^1–3^

The absorption of oral LT4 occurs in the small intestine and several factors, including medications, may interfere with it, thereby necessitating LT4 dose adjustments and more careful monitoring.^4^

In the stomach, the degree and rate of LT4 dissolution is essential for efficient absorption and relates directly to gastric acidity.^5^ *In vitro* studies have shown that an increase in medium pH affects the dissolution of LT4 tablets^6^ and similarly, an increase in gastric pH may affect dissolution and subsequent LT4 absorption.^7^

Drugs that block the secretion of gastric acid, such as proton pump inhibitors (PPIs), cause hypochlorhydria, and affect thyroid hormone (TH) parameters. Omeprazole treatment was associated with an increase in serum thyrotropin (TSH) levels in 10 patients treated with LT4 tablets, an effect that was reversed by an increase in the LT4 dose by 37%.

The daily requirement of LT4 was higher in patients with *Helicobacter pylori*–related gastritis, atrophic gastritis, or both conditions than in a reference group.^8^ Similarly, co-administration of lansoprazole was associated with a rise in TSH levels in 37 patients with primary hypothyroidism receiving LT4 replacement therapy, with 19% of the patients requiring a mean increase of 35% in LT4 dose after lansoprazole therapy was initiated.^9^ Epidemiologic studies demonstrated a significant interaction between LT4 and PPIs^10^ possibly representing the basis for an increased number of LT4 prescriptions during exposure to potentially interacting drugs.^11^

In addition to body mass index (BMI), gastric pH has been determined to be the most important independent variable in determining the effective dose of LT4 when administered in tablets.^12^

A soft gelatin capsule LT4 form (Tirosint^®^, manufactured by IBSA Institut Biochimique SA, Lugano, Switzerland, including also Tiche, Tcaps, Syntroxine), in which LT4 is dissolved in glycerin and surrounded by a gelatin shell, appears to be less sensitive to gastric pH variations. *In vitro* tests have demonstrated that the dissolution profile of the soft capsule is not influenced by pH of the medium.^6^ A study in 37 patients with impaired gastric acid secretion demonstrated that the therapeutic goal of LT4 replacement was maintained by doses of LT4 soft capsules that were 17% lower than those required for tablets.^13^

While LT4 soft capsules were bioequivalent to tablets in healthy subjects, two bioavailability studies in healthy volunteers treated with 600 μg of LT4 in single dose have shown that an intravenous infusion of 80 mg esomeprazole affected LT4 absorption to a lesser extent when it was given in soft capsules compared with standard tablets.^14^ Other findings suggest that LT4 soft capsule dose may be independent from actual gastric pH.

Following the switch from tablet to soft capsule in 28 patients with hypothyroidism on stable LT4 tablet doses, the LT4 requirement was the same in all 17 patients with normal gastric pH (median pH: 1.52), while in patients with high gastric pH (median pH: 5.02), the requirement for LT4 soft capsules was significantly reduced in 10 out of 11 patients.^15^

Based on this background, the present study in totally thyroidectomized patients on treatment with PPIs was designed to evaluate the effects of a switch from the standard LT4 tablet form to the same dose of LT4 soft capsules.

## Materials and Methods

The study population included adult patients with a history of hypothyroidism due to total thyroidectomy, on LT4 tablet (different brands and generics) dose ≥88 and ≤250 μg/day stable for at least 6 weeks and TSH at screening ≥0.3 and ≤4.0 mIU/L. To be eligible, participants were required to have a history of gastroesophageal reflux disease (GERD) or associated gastrointestinal issues treated chronically with prescription doses of PPIs.

Patients with any of the following conditions were excluded from participation: history of malabsorption, gastric bypass surgery, short-gut syndrome, inflammatory bowel disease, and other conditions of the gastrointestinal tract that could affect drug absorption; parenteral or assisted enteral feeding; presence of any medical condition that could affect safety of the patient or reliability of data; pregnancy; breast-feeding; regular consumption of foods known to affect LT4 absorption.

The study plan included a 4- to 6-week run-in period, during which eligible patients continued taking LT4 and PPIs as prescribed. If a subject required LT4 dose adjustment, it was possible to re-screen the patient until TSH normalization was achieved. At the end of the run-in period (baseline), patients were switched to treatment with Tirosint, at the same dose under which they were currently being treated. Soft capsules were taken for 12 weeks (treatment period), with an intermediate assessment of THs after 6 weeks.

During the entire study (run-in and treatment), LT4 was to be taken as a single daily dose in the morning, on an empty stomach, 1 hour before breakfast, separated by at least 4 hours from drugs, and at least 1 hour from foods known to interfere with LT4 absorption, such as soybean and derivatives, and dietary fibers. Intake of PPI was continued according to individual prescription and habits. Samples of peripheral venous blood were taken at each visit at around 8 a.m. ± 2 hours in fasting conditions and before LT4 dose.

No LT4 dose adjustments were allowed unless post-baseline TSH levels were >10 or <0.01 mIU/L or if this was considered necessary for the safety of the patient. Changes in the dose of PPI were acceptable during the study provided that the dose remained ≥20 mg/day for omeprazole, esomeprazole, and rabeprazole, ≥15 mg/day for lansoprazole, ≥30 mg/day for dexlansoprazole, and ≥40 mg/day for pantoprazole.

Use of other THs, as well as drugs affecting absorption/metabolism of thyroxine (T4), conversion of T4 to triiodothyronine (T3), THs serum transport, as well as tricyclic/tetracyclic antidepressants and tyrosine kinase inhibitors was not permitted.

Serum TSH levels were the primary endpoint of the study. The secondary efficacy endpoints were: serum levels of free thyroxine (fT4), total thyroxine (TT4), free triiodothyronine (fT3), total triiodothyronine (TT3), creatine-phosphokinase (CPK), sex-hormone binding globulin (SHBG), ferritin, angiotensin converting enzyme (ACE), and lipid profile.

Laboratory determinations were centralized at Q2 Solutions (Valencia, CA). Levels of all efficacy endpoints measured after changes in LT4 dose were not considered for the analysis. LT4 dose adjustments were evaluated both as number of patients requiring changes in daily dose and as mean change in daily dose from baseline to the end of the treatment period.

Adverse events (AEs), changes in vital signs (systolic and diastolic blood pressure, heart rate), and body weight were the safety endpoints. Clinically significant new or worsened clinical signs/symptoms of hypothyroidism or hyperthyroidism were to be reported as AEs. Additional information is provided in the Supplementary Methods.

The study protocol was approved by Institutional Review Boards before study start (Approval No.: Medstar Health Research Institute 2016-217 and 2017-069; Brany 18-02-354-05H; WIRB 420180437). Patients gave written informed consent to study participation. The research was completed in accordance with the Declaration of Helsinki. The study was registered in clinicaltrials.gov (NCT03094416).

A sample size of 48 subjects was considered to have 70% power to detect a difference in means of TSH of 0.7 mIU/L, assuming a standard deviation (SD) of differences of 1.9, using a paired *t*-test with a 0.050 two-sided significance level. Assuming a drop-out rate of 20%, a maximum number of 60 subjects included in the treatment period was expected to be needed.

All efficacy endpoints were analyzed in the intent-to-treat (ITT) set, which included all patients who received at least one dose of the study drug, and in the per-protocol (PP) set, which included all ITT patients who completed the study without any major protocol deviation.

Analysis of covariance (ANCOVA) models (with mixed-models repeated measures applied when patient count was ≥20% of ITT set), with age, sex, BMI, and the prescribed LT4 dose at baseline as covariates, were used in the analysis of changes from baseline in laboratory endpoints. An ANCOVA model, with LT4 daily dose/kg at each time point as dependent variable, was used in the analysis of changes from baseline to week 12 in mean LT4 daily dose.

Normal distributions for each dependent variable were tested with Shapiro Wilks: non-normally distributed dependent variables were log transformed. Analysis of efficacy endpoints in the ITT set was performed both on observed data and using the last observation carried forward (LOCF) approach for missing data. The AEs and vital signs were analyzed descriptively.

## Results

**FIG. 1.**
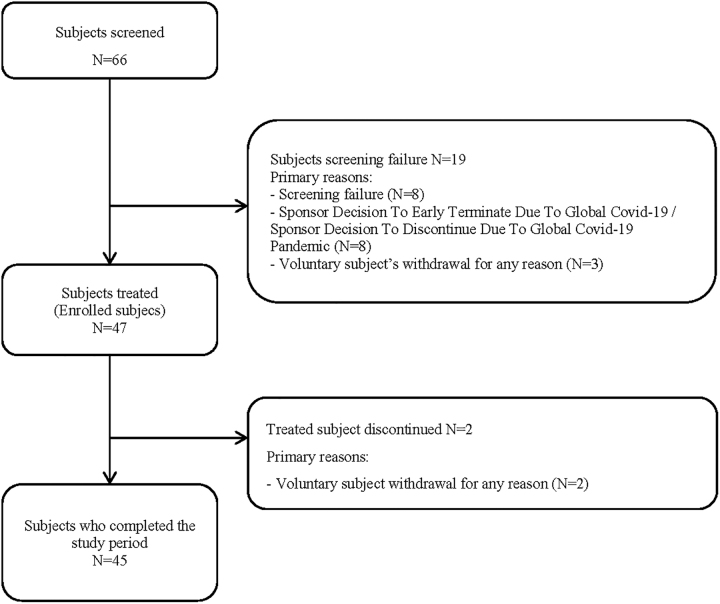
Patients' flow diagram.

Sixty-six patients were screened in 7 sites (3 academic hospitals, 4 private practices), 47 patients were enrolled and treated (ITT set), and 45 of them completed the study (2 patients withdrew consent) (Fig. 1). The planned target number of 48 completed subjects could not be reached due to COVID-19 pandemic outbreak. The PP set included 33 patients.

Fourteen patients (29.8% of ITT set) had major protocol deviations (some patients had more than one), which consisted of poor adherence to treatment (eight patients), use of prohibited medications (four patients), LT4 dose change or withdrawal from the study before week 6 (three patients), incorrect dosing of study treatment (two patients), and deviation from entry criteria (one patient).

Out of the 66 screened subjects undergoing treatment with LT4 tablets and PPIs, 27 (40.9%) had TSH values outside the reference range ≥0.3 and ≤4.0 mIU/L and required LT4 dose adjustment and re-screening. Two subjects (3.0%) still had TSH >4.0 mIU/L despite undergoing re-screening and LT4 dose adjustment twice during run-in. Table 1 shows the demographic characteristics of the 47 enrolled patients and their TH levels at screening.

**Table 1.** Demographic Characteristics and Thyroid Hormone Levels at Screening (Intent-to-Treat Set)

Variable	*n* = 47
Age, years	
Mean (SD)	55.38 (11.11)
Median (range)	57.0 (27 to 73)
Sex, *n* (%)	
Female	36 (76.60)
Male	11 (23.40)
BMI, kg/m^2^	
Mean (SD)	32.35 (8.30)
Median (range)	31.35 (15.40 to 54.00)
Prescribed daily LT4 dosage, μg	
Mean (SD)	144.54 (44.37)
Median (range)	137.0 (88.0 to 250.0)
Prescribed daily LT4 dosage, μg/kg	
Mean (SD)	1.65 (0.38)
Median (range)	1.60 (0.90 to 2.52)
TSH, mIU/L [0.4–4.5]	
Mean (SD)	1.64 (3.12)
Median (range)	0.67 (0.01 to 20.24)
fT3, pmol/L [3.5–6.5]	
Mean (SD)	4.31 (0.77)
Median (range)	4.30 (2.30 to 6.90)
fT4, pmol/L [10.3–23.2]	
Mean (SD)	16.58 (3.44)
Median (range)	16.70 (7.70 to 28.30)

Values in square brackets indicate the reference ranges.

BMI, body mass index; fT3, free triiodothyronine; fT4, free thyroxine; LT4, levothyroxine sodium; *n*, number of patients; SD, standard deviation; TSH, thyrotropin.

The ITT set included more females (36, 76.6%) than males (11, 23.4%). All patients had hypothyroidism due to total thyroidectomy and gastrointestinal disorders (45 GERD, and 2 gastritis) requiring treatment with PPIs. The most common PPIs were omeprazole (30 patients, 63.8%) and pantoprazole (10 patients, 21.3%).

**FIG. 2.**
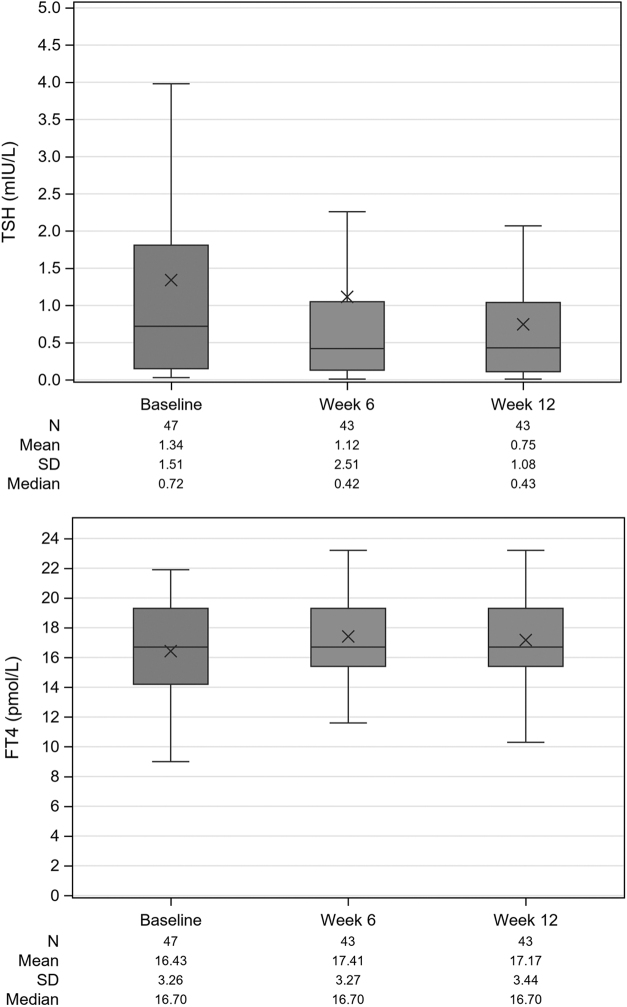

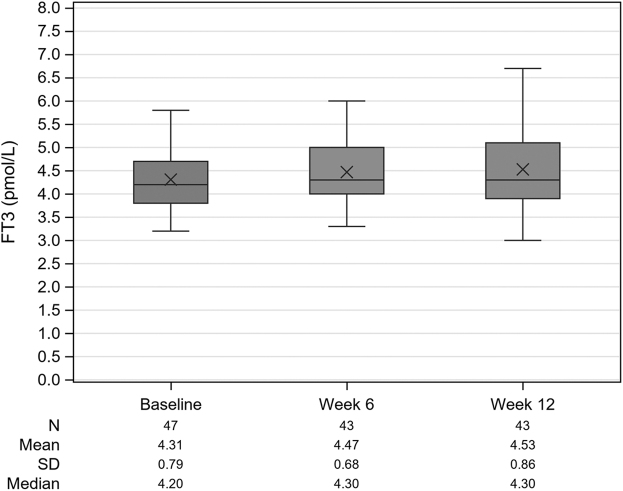
Box plot of TSH, fT4, and fT3 over time (ITT set, observed data). fT3, free triiodothyronine; fT4, free thyroxine; ITT, intent-to-treat; *N*, number of patients; SD, standard deviation; TSH, thyrotropin.

Table 2 shows the results of the primary endpoint in the ITT and PP sets. Mean TSH levels significantly decreased from baseline to weeks 6 and 12. The extent of the decrease at week 12 in the ITT set (Fig. 2) was higher in the analysis of observed data than with the LOCF (mean change at week 12: −0.68 mIU/L, *p* < 0.0001 and −0.30 mIU/L, *p* = 0.0003, respectively), as well as decrease from baseline was higher in the PP set (mean change at week 12: −0.80 mIU/L, *p* < 0.0001 for both observed data and LOCF) than in the ITT set.

**Table 2.** Results of Primary Efficacy Endpoint (Thyrotropin, mIU/L) in the Intent-to-Treat (Observed Data and Last Observation Carried Forward) and Per-Protocol Sets (Observed Data)

	*N*	Mean (SD)	Median (range)
ITT set			
Observed data			
Baseline	47	1.34 (1.51)	0.72 (0.03 to 5.73)
Week 6	43	1.12 (2.51)	0.42 (0.01 to 15.96)
Week 12	43	0.75 (1.08)	0.43 (0.01 to 6.35)
Change vs. baseline			
Week 6	43	−0.32 (2.80)	−0.32 (−5.62 to 15.22)^a^
Week 12	43	−0.68 (1.32)	−0.37 (−5.35 to 1.19)^b^
LOCF			
Baseline	47	1.34 (1.51)	0.72 (0.03 to 5.73)
Week 6	47	1.05 (2.41)	0.37 (0.01 to 15.96)
Week 12	47	1.05 (2.45)	0.43 (0.01 to 15.96)
Change vs. baseline			
Week 6	47	−0.29 (2.68)	−0.10 (−5.62 to 15.22)^a^
Week 12	47	−0.30 (2.64)	−0.09 (−5.35 to 15.22)^b^
PP set			
Observed data			
Baseline	33	1.55 (1.63)	0.87 (0.07 to 5.73)
Week 6	32	0.87 (1.06)	0.45 (0.01 to 4.59)
Week 12	33	0.75 (1.16)	0.43 (0.01 to 6.35)
Change vs. baseline			
Week 6	32	−0.72 (1.54)	−0.34 (−5.62 to 1.92)^b^
Week 12	33	−0.80 (1.43)	−0.46 (−5.35 to 1.19)^b^

^a^ *p* < 0.01, ^b^*p* < 0.001 vs. baseline in the ANCOVA model.

ANCOVA, analysis of covariance; ITT, intent-to-treat; LOCF, last observation carried forward; PP, per-protocol.

At week 12 (observed data and LOCF, ITT set), the ANCOVA models showed statistically significant effects of prescribed LT4 dosage at baseline (*p* = 0.0201 and *p* = 0.0230), whereas no statistically significant effects were observed for other covariates (age, BMI, sex). In the PP set, no covariates resulted in statistically significant change at week 12. A clinically significant (i.e., causing either entry or exit from laboratory reference ranges) decrease of TSH from baseline to week 12 was recorded in 11 (25.6%) of ITT subjects.

The results of the other TH parameters in the ITT set are shown in Table 3. The mean values of fT3, TT3, fT4, and TT4 increased from baseline to week 12 and were statistically significant for fT3 (mean change 0.26 pmol/L, *p* = 0.0062) and TT3 (mean change 0.10 nmol/L, *p* = 0.0438), whereas mean changes from baseline to week 12 of fT4 (0.59 pmol/L) and of TT4 (2.56 nmol/L) were not statistically significant (Fig. 2).

**Table 3.** Results of the Other Thyroid Hormone Parameters (Intent-to-Treat Set, Observed Data)

	*n*	Mean (SD)	Median (range)
fT3, pmol/L [3.5–6.5]			
Baseline	47	4.31 (0.79)	4.20 (3.20 to 7.50)
Change vs. baseline			
Week 6	43	0.17 (0.64)	0.20 (−1.50 to 2.10)^a^
Week 12	43	0.26 (0.57)	0.30 (−0.80 to 1.80)^b^
TT3, nmol/L [0.9–2.8]			
Baseline	47	1.40 (0.30)	1.30 (0.80 to 2.20)
Change vs. baseline			
Week 6	43	0.12 (0.30)	0.10 (−0.40 to 0.90)^b^
Week 12	43	0.10 (0.32)	0.10 (−0.50 to 0.90)^a^
fT4, pmol/L [10.3–23.2]			
Baseline	47	16.43 (3.26)	16.70 (9.00 to 21.90)
Change vs. baseline			
Week 6	43	0.81 (2.82)	0.00 (−3.90 to 7.70)^a^
Week 12	43	0.59 (2.74)	1.30 (−5.10 to 6.40)
TT4, nmol/L [62–134]			
Baseline	47	112.51 (30.88)	108.00 (40.00 to 188.00)
Change vs. baseline			
Week 12	43	2.56 (27.24)	3.00 (−63.00 to 67.00)

Values in square brackets indicate the reference ranges.

^a^ *p* < 0.05, ^b^*p* < 0.01 vs. baseline in the ANCOVA model.

TT3, total triiodothyronine; TT4, total thyroxine.

In the PP set, mean increases from baseline to week 12 in all TH parameters were higher than in the ITT population (0.86 pmol/L for fT4, 4.27 nmol/L for TT4, 0.29 pmol/L for fT3, and 0.12 nmol/L for TT3). Among covariates, fT3 was also associated with sex (*p* = 0.0055), fT4 with age (*p* = 0.0102), and TT4 with age (*p* = 0.0013) and sex (*p* = 0.0043).

The results of the other laboratory endpoints (Table 4) showed a statistically significant decrease from baseline to week 12 in mean levels of CPK (mean change −35.1 U/L, *p* = 0.0254), whereas there were no relevant changes in ferritin, SHBG, and ACE.

**Table 4.** Results of the Other Laboratory Secondary Efficacy Endpoints (Intent-to-Treat Set, Observed Data)

	*n*	Mean (SD)	Median (range)
CPK, U/L			
Baseline	46	119.07 (115.98)	92.00 (17.00 to 799.00)
Change vs. baseline			
Week 12	41	−35.07 (125.15)	−17.00 (−749.00 to 122.00)^a^
SHBG, nmol/L			
Baseline	47	54.47 (36.79)	40.00 (12.00 to 154.00)
Change vs. baseline			
Week 12	43	0.88 (19.19)	2.00 (−56.00 to 77.00)
Ferritin, μg/L			
Baseline	47	64.64 (58.53)	40.00 (4.00 to 243.00)
Change vs. baseline			
Week 12	43	−6.44 (30.24)	−1.00 (−102.00 to 86.00)
ACE, U/L			
Baseline	44	32.43 (27.06)	25.50 (5.00 to 165.00)
Change vs. baseline			
Week 12	38	−0.40 (11.62)	0.50 (−55.00 to 18.00)
Triglycerides, nmol/L			
Baseline	45	1.58 (1.00)	1.30 (0.58 to 5.82)
Change vs. baseline			
Week 12	40	0.08 (0.54)	0.00 (−1.20 to 2.06)
LDL-C, nmol/L			
Baseline	43	2.60 (0.84)	2.53 (0.79 to 5.43)
Change vs. baseline			
Week 12	39	−0.21 (0.56)	−0.11 (−2.33 to 0.39)^a^
HDL-C, nmol/L			
Baseline	45	1.49 (0.38)	1.45 (0.55 to 2.45)
Change vs. baseline			
Week 12	40	−0.03 (0.25)	0.00 (−0.75 to 0.70)
VLDL-C, mnmol/L			
Baseline	43	0.64 (0.27)	0.58 (0.27 to 1.22)
Change vs. baseline			
Week 12	39	0.05 (0.23)	0.01 (−0.37 to 0.95)
Total cholesterol, mnmol/L			
Baseline	45	4.80 (0.85)	4.75 (3.15 to 7.70)
Change vs. baseline			
Week 12	40	−0.20 (0.67)	−0.08 (−2.95 to 0.85)^a^

^a^ *p* < 0.05 vs. baseline in the ANCOVA model.

ACE, angiotensin converting enzyme; CPK, creatine-phosphokinase; HDL-C, high-density lipoprotein-cholesterol; LDL-C, low-density lipoprotein-cholesterol; SHBG, sex-hormone binding globulin; VLDL-C, very-low-density lipoprotein-cholesterol.

The results of lipid profile showed a statistically significant decrease of low-density lipoprotein-cholesterol (LDL-C; mean change −0.21 nmol/L, *p* = 0.0203) and total cholesterol (mean change −0.20 nmol/L, *p* = 0.0468), whereas no significant changes were reported for triglycerides, high-density lipoprotein-cholesterol, and very-low-density lipoprotein-cholesterol.

Only two patients in the ITT set changed the dosage during the treatment period, and the mean prescribed LT4 dosage remained unchanged from baseline to week 12 (mean dosage: 142.0 μg/1.62 μg/kg and 141.1 μg/1.60 μg/kg, respectively).

The mean (SD) overall compliance to soft capsules was 95.3 (15.0) % of scheduled and was considered excellent in 36 patients (76.6%), good in 2 (4.3%), and poor in 7 (14.9%).

Eleven patients (23.4%) reported AEs during run-in treatment with LT4 tablets and PPIs. Fourteen patients (29.8%) reported AEs during treatment with soft capsules and PPIs, which were considered related to treatment only in one patient, who had gastroenteritis. No cases of signs or symptoms of hypothyroidism or hyperthyroidism were reported.

One patient had a non-fatal serious AE (asthma exacerbation) during the treatment period, which was considered not related to treatment, while two patients had four non-fatal serious AEs (bradycardia, bronchitis, urinary tract infection, and dehydration) during the run-in period.

The mean values of vital signs and body weight remained unchanged from baseline up to week 12, and there were no clinically significant individual changes for any parameter.

## Discussion

Polypharmacy is considered a major health problem that particularly affects the elderly.^16^ PPIs are among the most prescribed drugs worldwide and there is growing concern about their long-term use and appropriateness of prescription, in relation to the emerging evidence of adverse effects. A recent study analyzed data of community-dwelling people aged 65 years and older, who were prescribed PPIs for GERD and found that only 41.2% of patients received appropriate prescriptions.^17^ A more rational use of PPIs is, therefore, recommended.^18^

PPIs are known to interfere with the absorption of drugs, such as LT4, posing further concern about polypharmacy in hypothyroid patients.^19,20^ The recommended management is additional patient's monitoring, with check of THs parameters and subsequent LT4 tablet dose adjustments.

This approach is not always practicable, because the duration of PPI use is highly variable, as PPIs are often used intermittently (10% of long-term users)^18,21^ and most of the times beyond the control of the endocrinologist. Moreover, differences in excipients and manufacturing among generics LT4 tablets pose an additional level of complexity in the interaction between LT4 and PPI therapy.

To our knowledge, this is the first study to investigate the effects of a switch from LT4 tablet to LT4 soft capsule on the hormonal and biochemical parameters of thyroidectomized patients receiving concomitant treatment with PPIs.

In this study, thyroidectomized patients were selected due to the absence of endogenous production of THs and therefore highest sensitivity to changes in the exogenous supplementation of LT4. A daily dose of LT4 at screening ranging between 88 and 250 μg was selected because patients who undergo thyroidectomy are usually treated with dosages above 88 μg, while patients in treatment with unusually high doses of LT4 may have other underlying factors affecting absorption of the drug.

The results indicate that treatment with soft capsules was effective in improving TH parameters in thyroidectomized patients taking both LT4 and PPIs, with a significant decrease in TSH from baseline up to weeks 6 and 12 in the ITT population. The effect was even more marked in the PP set: this is not an unexpected finding, considering that most protocol deviations were due to poor adherence, incorrect dosing, or use of prohibited medications, which might have limited the effects of soft capsules. The LOCF analysis of TSH showed less marked changes at week 12, due to the carrying forward of baseline data of four patients not being evaluated at week 12.

The effects observed on TSH were consistent with those noted on THs, which increased from baseline to week 12 (statistically significant for fT3 and TT3).

The effects on lipid profile, with LDL-C and total cholesterol decreasing significantly, are an expected finding in case of normalization of hypothyroidism, in view of the known link between thyroid status and cholesterol levels.^22^

A marked rise in CPK levels has been reported to be associated with increased TSH levels in patients with hypothyroidism^23^ and thus the reported lower CPK levels on switch to soft capsules further support the improved metabolic state that paralleled the effects on THs.

Only one patient required a dose increase following an observed increased TSH value at week 6, which was related to poor adherence to treatment. There were no instances of dose adjustment related to TSH suppression or over treatment. Considering that a significant proportion of patients (up to 75%) require LT4 adjustment following thyroidectomy,^24^ the evidence that <5% of patients required changes in LT4 dosage suggests that adequate metabolic control was maintained after switch and that there were no safety issues related to the switch. In line with the known safety profile of the LT4 soft capsules,^25^ treatment was well tolerated, with no cases of hypo- or hyperthyroidism recorded.

This study has several limitations. First, it did not include a control arm on LT4 tablets alone. Comparisons with the standard tablet form were determined versus the baseline values measured at the time of the switch from LT4 tablets. The inclusion of a control group maintained on treatment with tablets may have allowed a more reliable comparison between the two forms not only in terms of effects on TH levels, but also in terms of optimization of therapy and requirements for dose changes. To minimize potential bias due to lack of a control arm, baseline values were obtained after a run-in period with LT4 tablets and PPIs, to stabilize the patients under the same study conditions used during the treatment period (particularly referring to proper drug administration and restrictions).

Second, the run-in was conducted using LT4 tablets purchased by patients from their local pharmacies. Thus, it was not possible to compare the content in LT4 and T3 impurity among the several batches of LT4 tablets and LT4 capsules used in the study.

A third limitation was the relatively low number of participants, with the COVID-19 outbreak causing the inability to reach the planned number of 48 completed subjects and ∼30% of patients excluded from the PP set due to major deviations (mostly, poor adherence to treatment). Nevertheless, the analysis conducted on the two populations (ITT and PP) provided similar results. A higher number of patients would not only increase robustness of results, particularly for secondary endpoints, but also allow more reliable estimation of the effects of the examined covariates on primary and secondary efficacy endpoints or subgroup analyses. It should be noted that the *a-posteriori* power calculation on results for primary endpoint on original data gave a higher study power of 91%.

Fourth, it was not possible to determine the increase in TSH on the initiation of PPI treatment in the study patients, because the therapy with LT4 tablets and PPIs was already ongoing at the time of recruitment. Interestingly, though, the decrease in TSH measured after the switch from LT4 tablet to soft capsules is consistent with the increase in TSH reported by other authors on start of PPI therapy in LT4 treated patients. In a group of 37 patients with hypothyroidism and normal TSH levels receiving LT4 replacement therapy and in whom lansoprazole therapy was initiated, the mean change in TSH from before to at least 2 months after initiation of PPI therapy was 0.69 mIU/L (*p* = 0.035).^9^

Fifth, the inclusion age, weight, and LT4 dose/kg ranges were wide. This was a decision driven by the interest in representing real-life conditions and the general population. Nonetheless, the confounder related to patients' selection was minimized by having each patient serve as their own control and by considering the variables age, BMI, and prescribed LT4 dosage at baseline as covariates in the statistical models.

Sixth, the variability in the time between tablet or capsule ingestion and breakfast (30–60 minutes) may have contributed to some variability in the results. However, because this was a phase IV study, it was bound to respect the conditions prescribed in the products label and, also, tighter instructions may have resulted in higher non-adherence.

Finally, although a period of 12 weeks can be considered adequate for stabilization of TSH following a change of LT4 therapy, the observation period of treatment with soft capsules was relatively short and preliminary findings of this study should be confirmed over a longer-term exposure.

## Conclusions

In conclusion, significant lowering of TSH was seen in a cohort of patients with hypothyroidism on stable treatment with LT4 and PPIs when switched from LT4 tablets to an equivalent LT4 dose given as soft capsules (Tirosint). These observations suggest that LT4 absorption may be less affected by PPIs when given in the form of soft capsules.

## Supplementary Material

Supplemental data
