# Supplementary material for: Efficacy of Levothyroxine Sodium Soft Gelatin Capsules in Thyroidectomized Patients Taking Proton Pump Inhibitors: An Open-Label Study
Source: Thyroid. 2023 Dec 7;33(12):1414–22. doi: 10.1089/thy.2023.0382 (PMC10754356; doi:10.1089/thy.2023.0382)
Supplement: Supplemental data [file Suppl_Methods.docx]

**METHODS SUPPLEMENT**

At each study visit, the Investigator had to dispense a Patient Diary and instruct the subject on how to complete the diary and how to comply with the study requirements (i.e. drugs intake, study restrictions, etc.).

Subjects were asked to record their daily levothyroxine tablet / Tirosint^®^ and PPI intake in the diary, starting from the first visit (Screening) until the end of the study.

The following instructions were provided to study subjects at each visit, starting from Screening until the end of the study, written in the diary:

Run-In Phase

“Please remember that your levothyroxine sodium medication should be taken as a single daily oral dose in the morning on an empty stomach, one-half to one hour before breakfast. It should be taken at least 4 hours before or after drugs known to interfere with its absorption (i.e. iron and calcium supplements, bile acid sequestrants and ion exchange resins) and at least 1 hour apart from foods known to interfere with its absorption (e.g. soybean and derivatives, cottonseed meal, walnuts, and dietary fiber).

The morning of the next visit, you will take your daily levothyroxine dose only after blood sampling.”

Treatment Phase

“Please remember that Tirosint^®^ should be taken as a single daily oral dose in the morning on an empty stomach, one-half to one hour before breakfast. It should be taken at least 4 hours before or after drugs known to interfere with its absorption (i.e. iron and calcium supplements, bile acid sequestrants and ion exchange resins) and at least 1 hour apart from foods known to interfere with its absorption (e.g. soybean and derivatives, cottonseed meal, walnuts, and dietary fiber).

The morning of the next visit, you will take your daily Tirosint^®^ dose only after blood sampling.”

In addition, from Screening to end of study, subjects also received the following instructions for the next visit:

- “be fasting for 10 hours before coming to the clinic,
- do not take your daily dose of levothyroxine,
- do not take biotin during the 2 days before the visit,
- bring this diary with you.”

Samples of peripheral venous blood were to be taken from the patient at each visit at around 8h00 ± 2h. The Investigator had to record if the blood sample was taken pre-LT4 dose or post-LT4 dose and, in the latter case, this was reported as a deviation from the study procedures.

The following assays (methodology and analyzer) were used for the analysis of laboratory parameters:

TSH, FT4, TT4, FT3, TT3: Immunoassay with Siemens ADVIA Centaur

ACE: Spectrophotometry with Beckman-Coulter AU2700

CPK: Kinetic with Olympus AU 640/2700/5400

Ferritin: Immunochemiluminometric Assay with Siemens ADVIA Centaur

Lipid panel: Spectrophotometry with Olympus AU 640/2700/5400

SHBG: Immunoassay with Siemens Immulite 2000
